# Comorbidity of Alcoholism and Psychiatric Disorders: An Overview

**Published:** 2002

**Authors:** Ismene L. Petrakis, Gerardo Gonzalez, Robert Rosenheck, John H. Krystal

**Affiliations:** Ismene L. Petrakis, M.D., is an associate professor of psychiatry; Gerardo Gonzalez, M.D., is an assistant professor of psychiatry; Robert Rosenheck, M.D., is a professor of psychiatry and public health; and John H. Krystal, M.D., is the Albert E. Kent Professor and deputy chairman for research in psychiatry, all at the Yale University School of Medicine, New Haven, Connecticut. Robert Rosenheck, M.D., is also director of the VA Northeast Program Evaluation Center, West Haven, Connecticut

**Keywords:** AODD (alcohol and other drug dependence), comorbidity, mentally ill, prevalence, health care utilization, drug therapy, antidepressants, serotonin uptake inhibitors, psychosocial treatment method, antipsychotic tranquilizers, 12-step program

## Abstract

People with alcohol use disorders often have co-occurring psychiatric disorders, but they frequently do not receive specialized substance abuse treatment that addresses both conditions. Although pharmacological and psychosocial treatments for alcohol use disorders and psychiatric disorders can be integrated to help these patients, relatively few clinical studies have tested these types of treatments. As mental health and substance abuse facilities expand their services for patients with dual disorders, further research is needed to guide the treatment of this patient population.

Alcohol abuse and dependence frequently occur with other psychiatric conditions; this dual diagnosis is called comorbidity. Professionals working with comorbid patients face unique and challenging dilemmas about how to provide the best treatment to address both conditions. Despite growing interest in these problems, relatively few clinical studies have tested treatments for this patient population. This review examines the prevalence of alcohol abuse and dependence with other psychiatric disorders and the patterns of treatment among comorbid patients. It also describes how treatment approaches can be integrated for patients with these comorbid disorders and offers suggestions for future directions in treatment research.

## Rates of Psychiatric Disorders in Patients with Alcohol Use Disorders

The two main U.S. studies that have addressed the epidemiology of comorbidity are the National Comorbidity Survey (NCS) (Kessler et al. 1996) and the Epidemiologic Catchment Area (ECA) study (Regier et al. 1990). The NCS was a nationally representative household survey of people ages 15–54 conducted in 1990–1991. Diagnoses were based on the results of diagnostic interviews. The ECA study reflected data from the U.S. general population as well as an institutionalized population, using data from the National Institute of Mental Health Epidemiologic Catchment Area Program. A total of 20,291 people ages 18 and older were interviewed between 1980 and 1984.

Alcohol abuse is an alcohol use disorder characterized by continued drinking despite negative consequences and the inability to fulfill responsibilities. Alcohol dependence, also known as alcoholism, is characterized by a craving for alcohol, possible physical dependence on alcohol, an inability to control one’s drinking on any given occasion, and an increasing tolerance to alcohol’s effects (American Psychiatric Association [APA] 1994). The table shows the prevalence rates of psychiatric disorders among the respondents to the NCS and the ECA study who were diagnosed with alcohol abuse or dependence and a comorbid psychiatric disorder.

### Alcohol Abuse

In the NCS, 2.5 percent of the respondents were classified as having abused alcohol but not as having been alcohol dependent during the 12-month period before the survey. In the ECA study, 3.5 percent of respondents were diagnosed as having alcohol abuse at some point in their lifetime. Among respondents to the NCS who had abused alcohol, 12.3 percent also met the criteria for a mood disorder (including major depression and bipolar disorder, characterized by shifts in mood between depression and manic episodes) during the previous year. Of those with comorbid mood disorders, 11.3 percent had major depressive disorder and 0.3 percent had bipolar disorder. (For each category of comorbid disorders, the prevalences of only a few specific disorders are reported. Therefore, the prevalence rates of the specific disorders [e.g., major depressive disorder, bipolar disorder] do not total the rate for the general type [e.g., mood disorders]). Post-traumatic stress disorder (PTSD) was the most frequently occurring anxiety disorder (i.e., compared with generalized anxiety disorder [GAD] and panic disorder), affecting 5.6 percent of respondents diagnosed as alcohol abusers. The estimated rates of GAD and panic disorder were smaller and similar to each other (1.4 percent and 1.3 percent, respectively). There were no statistically significant associations between alcohol abuse and any of these psychiatric disorders.

The lifetime rates for comorbid schizophrenia were available only from the ECA study. Almost 10 percent of the people diagnosed as alcohol abusers in that study also had a diagnosis of schizophrenia. The odds of having schizophrenia were 1.9 times higher among people who abused alcohol than among those who did not.

### Alcohol Dependence

For each of the psychiatric disorders examined, prevalence rates were higher among people diagnosed as alcohol dependent than among alcohol abusers (see table). In the NCS study, 7.2 percent of the survey respondents were diagnosed as alcohol dependent during the 12 months before the survey, and in the ECA study, 7.9 percent of respondents were diagnosed as having been alcohol-dependent at some point in their lifetime. Almost one-third (29.2 percent) of NCS respondents with alcohol dependence had a mood disorder. Alcohol-dependent respondents were 3.9 times more likely to have had major depressive disorder (27.9 percent) during the previous year than those who were not alcohol dependent. Although bipolar disorder was diagnosed in only 1.9 percent of people with alcohol dependence, the odds of having this disorder were 6.3 times greater among alcohol-dependent people compared with others.

**Table:** Prevalence of Psychiatric Disorders in People with Alcohol Abuse and Alcohol Dependence

	Alcohol abuse		Alcohol dependence	

Comorbid Disorder	1-year rate (%)	Odds ratio	1-year rate (%)	Odds ratio
**National Comorbidity Survey**^1^				
**Mood disorders**	12.3	1.1	29.2	3.6*
Major depressive disorder	11.3	1.1	27.9	3.9*
Bipolar disorder	0.3	0.7	1.9	6.3*
**Anxiety disorders**	29.1	1.7	36.9	2.6*
GAD	1.4	0.4	11.6	4.6*
Panic disorder	1.3	0.5	3.9	1.7
PTSD	5.6	1.5	7.7	2.2*

**Epidemiologic Catchment Area**^2^ **study**	Lifetime rate (%)	Odds ratio	Lifetime rate (%)	Odds ratio
Schizophrenia	9.7	1.9	24	3.8

NOTES:

* Odds ratio was significantly different from 1 at 0.05 level. The odds ratio represents the increased chance that someone with alcohol abuse or dependence will have the comorbid psychiatric disorder (e.g., a person with alcohol dependence is 3.6 times more likely to also have a mood disorder compared to a person without alcohol dependence).

The 1-year rate of a disorder reflects the percentage of people who met the criteria for the disorder during the year prior to the survey.

The lifetime rate reflects the percentage of people who met the criteria for the disorder at any time in their lifetime.

SOURCES:

1 Kessler et al. 1996.

2 Regier et al. 1990.

Furthermore, among people with alcohol dependence, an estimated 36.9 percent met the criteria for an anxiety disorder during the previous year. Of these, 11.6 percent had GAD, 3.9 percent had panic disorder, and 7.7 percent had PTSD. The chances of having comorbid anxiety disorders were significantly increased among those diagnosed as alcohol-dependent, with the exception of panic disorder. According to the ECA study, the lifetime rate of comorbid schizophrenia in the alcohol-dependent group was estimated to be 24 percent. A person with alcohol dependence was 3.8 times more likely to have schizophrenia than one without alcohol dependence.

The NCS found that the median age of onset for comorbid psychiatric disorders preceded the median age of onset for all addictive disorders by 10 years (11 years old compared with 21 years old). In addition, the majority of respondents who had both a psychiatric disorder and an addictive disorder reported that they had begun to suffer from at least one psychiatric disorder before the addictive disorder started. The one exception to this order of onset was that 72 percent of alcohol-abusing males reported that their alcohol use disorder preceded the onset of a mood disorder.

## Patterns of Health Service Utilization Among Comorbid Patients

Alcoholism is one of the most costly health care problems faced by society, with an estimated societal cost (which includes, for example, productivity costs associated with alcohol-related morbidity and mortality, treatment costs, and costs associated with alcohol-related crime and traffic crashes) of $184.6 billion dollars per year (Harwood et al. 2000).

Data from the NCS study (Wu et al. 1999) indicate that the probability of receiving specialized mental health services (i.e., services for psychiatric disorders not including substance use disorders) is higher for people with a comorbid psychiatric disorder and either alcohol abuse (78 percent) or alcohol dependence (41 percent) than it is for those who have alcohol abuse or dependence without a comorbid psychiatric disorder (25 percent and 24 percent, respectively). In comparison, the probability of attending a substance abuse treatment program is greater for alcohol abusing (19 percent) and alcohol-dependent (21 percent) people without comorbid psychiatric disorders than for those with such disorders (0 percent and 16.3 percent, respectively). The factors identified in the NCS that predicted an increased likelihood of service utilization (including both mental health and substance abuse treatment) among alcohol-dependent people with comorbid psychiatric disorders were: (1) being between 36 and 44 years old; (2) being divorced, widowed, or separated; (3) having an annual income of between $20,000 and $69,000; (4) having a positive family history for mental illness; and (5) having recent legal problems. This suggests that many people entering mental health or substance abuse treatment have other psychosocial issues that need to be addressed, and that many people with comorbid disorders are not receiving the specialized substance abuse care they need.

In a series of papers produced in the early 1990s, Holder and colleagues affirmed that alcoholism treatment can result in markedly reduced health care costs (Holder et al. 1991; Blose and Holder 1991). The high rates of comorbid mental disorders among people with alcohol abuse and dependence and the low rates of appropriate treatment among comorbid patients provide substantial evidence that integrated services are needed which can address both disorders. It is important for both mental health and substance abuse professionals to recognize the dual disorders and to treat them as such early in their course, because early intervention may reduce the severity of both disorders, improve the eventual outcome, and reduce health care costs.

## Treatment

Although there has been an explosion of research on strategies for treating alcohol-related disorders, most of this research has focused on treatment for patients with a single diagnosis of alcohol abuse or dependence and not on patients with a dual diagnosis. Likewise, well-established treatments for psychiatric disorders have, for the most part, not been evaluated in patients with co-occurring alcohol-related disorders.

As a result of the recent widespread recognition of the need for more effective treatments, however, more specialized treatment for comorbid patients is becoming available. Mental health and substance abuse facilities are expanding services for patients with dual disorders, integrating psychiatric and substance abuse treatments, and seeking broader education and training for the professionals who staff these facilities. A greater understanding of the psychological and neurobiological mechanisms at work in psychiatric and substance use disorders has shaped research in this area. Although they still represent a small fraction of the research, several studies conducted during the past 5 to 10 years have investigated the use of integrated treatment for comorbid alcoholic patients. These studies have shown not only that this research is feasible and productive but that it is useful for informing clinicians about potential effective treatments for this group of patients (e.g., Cornelius et al. 1997*a*,*b*). The following sections describe the various pharmacologic and psychosocial treatments available for treating alcohol use disorders alone and with comorbid psychiatric conditions.

### Pharmacologic Treatment

Several aspects of comorbidity suggest that medications may be particularly important for this group of alcoholics. First, some alcohol-dependent people with comorbid diagnoses face greater difficulties in accessing and using traditional alcoholism treatment and self-help groups in which many members do not have comorbid disorders. For example, people with pronounced paranoia or negative symptoms (e.g., social and emotional withdrawal) may be uncomfortable in a group setting or with a treatment approach that includes confrontation. Second, pharmacological treatments are generally familiar to dually diagnosed patients, because many already are used to taking medications for their psychiatric disorder, and dosage scheduling can be readily integrated into a medication schedule for the comorbid condition. Third, the cognitive symptoms common in comorbid mental disorders may undermine patients’ motivation and ability to learn new material in psychosocial treatments, particularly early in recovery. For example, patients with thought disorders or slowed thinking secondary to depression may have difficulty concentrating and learning or completing homework assignments in treatment that is based on cognitive behavioral therapy.

Although medications may be especially beneficial for comorbid patients for these reasons, clinicians need to be educated about medication interactions and compliance issues to use these treatment tools most effectively.

The first three medications discussed below—disulfiram, naltrexone, and acamprosate—are used to treat alcohol use disorders, and have rarely been evaluated in the treatment of comorbid patients. The next set of medications— antidepressants, antianxiety medications, and antipsychotic medications—are used to treat psychiatric disorders, and have been evaluated in some studies among alcoholics with comorbid conditions.

#### Disulfiram

Disulfiram, one of the two medications currently approved by the U.S. Food and Drug Administration (FDA) for the treatment of alcoholism, has been used clinically in the management of alcoholic patients for 50 years (Meyer 1989). If patients comply with a regimen of this medication, disulfiram can promote total abstinence from alcohol. Disulfiram works with the body’s normal breakdown of alcohol to produce a byproduct called acetaldehyde, which can be highly toxic, so that a person taking disulfiram who drinks alcohol will experience substantial aversive reactions, including vomiting, flushing, headache, and severe anxiety. These reactions are strong enough to make most people completely stop drinking while taking disulfiram at its prescribed dose. This result is disulfiram’s biggest strength and its greatest weakness— some people decide instead to stop taking disulfiram, and this lack of compliance may be the main reason disulfiram is not more effective. The practical importance of compliance was demonstrated by a large-scale Veterans’ Administration cooperative study (Fuller et al. 1986) in which disulfiram-and placebo-treated patients had similar outcomes, and compliance was the best predictor of positive outcome. Several studies on increasing compliance have shown the effectiveness of behavioral interventions, such as supervised administration, administration by a significant other, or establishing written agreements on taking disulfiram as prescribed (Chick et al. 1992; Chick 1999; O’Farrell and Bayog 1986). Some evidence suggests that disulfiram is effective in treating patients with comorbid alcohol and cocaine dependence (Carroll et al. 1998).

Clinical reports have suggested that disulfiram may precipitate several psychiatric symptoms, including delirium, depression, anxiety symptoms, mania, and psychosis (Larson et al. 1992). However, most of these reports chronicle situations that occurred before 1970, when dosages of 1 to 2 grams were used (compared with current doses of 250–500 mg), and the definitions of the psychiatric symptoms were not standardized. Aside from these early clinical reports, however, there are few studies on the use of disulfiram in patients with comorbid psychiatric disorders. In one study of disulfiram in patients with dual disorders (including schizophrenia, bipolar disorder, anxiety, and personality disorders) there were no reports of disulfiram worsening psychotic symptoms, so the researchers concluded that disulfiram could be a useful adjunct if the patient was receiving appropriate psychiatric medications (Kofoed et al. 1986). Some case reports indicated that disulfiram interacted with common psychiatric medications by slightly increasing the levels of some antipsychotic and antidepressant medications found in patients’ blood (Larson et al. 1992) A more thorough evaluation, however, suggests that little evidence exists of a clinically meaningful interaction between disulfiram and medications commonly prescribed to treat major psychiatric disorders, including antidepressants, antipsychotics, and antimanic agents (Larson et al. 1992). Nevertheless, because there are clinical reports that disulfiram may induce psychiatric symptoms, prescribing disulfiram for patients with psychiatric comorbidity, particularly those with a psychotic disorder, should be done with caution.

#### Naltrexone

Naltrexone, the other medication approved by the FDA for the treatment of alcoholism, is designed to block the action of a key chemical (i.e., opioid) in the brain (i.e., it is an opioid antagonist). After studies with laboratory animals suggested that naltrexone may be effective in treating alcohol dependence, it was evaluated in two now well-known clinical trials. Volpicelli and colleagues (1992) studied naltrexone as an adjunctive treatment to standard psychotherapy in a placebo-controlled, double-blind study of 70 recently detoxified alcoholic patients. The results showed that naltrexone-treated patients reported lower levels of alcohol craving, fewer drinks consumed per occasion, fewer drinking days, and lower rates of relapse to heavy drinking compared with placebo-treated patients. Volpicelli and colleagues’ initial findings were replicated and extended by O’Malley and colleagues (1992). In a double-blind, placebo-controlled study of 97 alcohol-dependent patients, the researchers found that naltrexone-treated patients had higher rates of continuous abstinence, fewer drinking days, and lower rates of relapse to heavy drinking. A third clinical trial of 131 recently abstinent alcohol-dependent people (Anton et al. 1999) demonstrated that naltrexone-treated patients had lower rates of relapse to heavy drinking and fewer drinking days and that naltrexone improved the therapeutic effects of cognitive behavioral therapy.

Studies using self-administration and retrospective patient reports from clinical trials have provided evidence for a psychological mechanism of action for naltrexone’s efficacy (Volpicelli et al. 1995; O’Malley et al. 1996; Davidson et al. 1999; McCaul et al. 2000). For example, three of naltrexone’s effects have emerged from human studies: (1) it reduces craving or enhances the ability to maintain abstinence; (2) it alters the positive reinforcement of drinking; and (3) it reduces the priming effect of taking an initial drink, making a relapse to heavy drinking less likely. In contrast with disulfiram, naltrexone use does not lead to a powerful aversive reaction if patients consume alcohol. Patients may be more willing to initiate naltrexone treatment and to continue to take the medication because they know that drinking is not prohibited. If they do take a drink, the reduced intoxication and diminished priming effect may prevent more extensive relapse. Despite naltrexone’s advantages over disulfiram in patient acceptance, compliance has been shown to be a major factor in its use as well (Volpicelli et al. 1997). Further, a recent large multisite trial in alcohol-dependent veterans failed to confirm any effect of naltrexone on drinking outcomes (Krystal et al. 2001), and its exact role in the treatment of alcoholism is not well-defined.

When considering using naltrexone and other opioid antagonists to treat patients with comorbid disorders, it is especially important to determine whether these types of medications affect psychosis, affective or anxiety symptoms, or symptoms of PTSD. A study of naltrexone found that it did not worsen the symptoms associated with schizophrenia (Sernyak et al. 1998). Another opioid antagonist, naloxone, was found to have a modest therapeutic effect on psychotic symptoms (Pickar et al. 1982). Although several studies have suggested that naloxone may attenuate some manic symptoms, this finding has not been replicated by other research (Pickar et al. 1982). Naloxone has been shown to have no significant mood effects in patients with depression (Terenius et al. 1977). Although case reports have suggested that naltrexone may precipitate panic attacks (Luby and Marrazzi 1987), a more rigorous evaluation has suggested that naloxone does not precipitate panic attacks either alone or when administered with sodium lactate (a respiratory stimulant that can elicit panic attacks) (Liebowitz et al. 1984). No controlled studies have evaluated the effects of opioid antagonists on PTSD.

Croop and colleagues (1995) studied the effects of naltrexone in more than 500 alcohol-dependent patients, including a large percentage of dually diagnosed patients simultaneously receiving medications for other comorbid mental disorders. The rate of adverse events in naltrexone-treated patients did not differ in patients with and without comorbid mental disorders. In another study, a chart review of 72 patients treated in an outpatient mental health clinic for major psychiatric illnesses, including schizophrenia, bipolar disorder, schizoaffective disorder, and comorbid alcohol dependence suggested that naltrexone can have a good clinical response as measured by treatment retention and alcohol consumption (Maxwell and Shinderman 2000).

Although this research offers promising evidence about the use of naltrexone in patients with comorbid psychiatric disorders, further research evaluating its efficacy, tolerability, and safety is warranted.

#### Acamprosate

Preclinical research on acamprosate suggests that it interacts with a receptor, or binding molecule, known as the *N*-methyl-d-aspartate (NMDA) receptor, for the brain chemical (i.e., neurotransmitter) glutamate. Interactions with other receptors are currently being studied. A series of placebo-controlled European studies involving more than 4,500 patients have indicated that detoxified patients treated with acamprosate were less likely to drop out of treatment and achieved higher rates of abstinence (Mason and Ownby 2000) than those on placebo. Researchers have hypothesized that acamprosate may help patients achieve abstinence at least partially by diminishing withdrawal symptoms (Mason and Ownby 2000). Although approval of acamprosate in the United States is still pending additional clinical trials, it is widely available in Europe. Additional studies will help to answer questions about the use of acamprosate in alcoholism treatment. Research establishing its efficacy was conducted in patients without comorbid psychiatric disorders.

#### Antidepressants

The relationship between depression and alcoholism is complex because of overlapping symptoms, common neurobiological abnormalities (Pettinati et al. 2000*a*), and similar treatments (pharmacological and psychosocial). Two classes of antidepressants—the selective serotonin reuptake inhibitors (SSRIs), which affect the production and/or absorption of the neurotransmitter serotonin, and the tricyclic antidepressants (TCAs)—have been evaluated in patients with comorbid depression and alcohol use. The TCAs, including desipramine and imipramine, have been found effective in treating depression in alcoholics (Kranzler and Rounsaville 1998), but there is no consistent evidence that they are effective in decreasing alcohol consumption. With these mixed results and considering the potential for overdose, the use of TCAs in alcohol-abusing patients may be unwise.

The SSRIs, because they have fewer serious side effects than the older antidepressants, have become the first line of treatment for depressive disorders (Berman and Charney 1999). Researchers have hypothesized that the SSRIs may directly affect alcohol consumption (e.g., by preventing relapse to alcohol use during stress) (Sellers et al. 1994). This hypothesis is supported by some preclinical data and by some clinical data, which have shown that SSRIs are effective in reducing alcohol use in depressed people but not in reducing alcohol use in people without depression. Several studies have shown that SSRIs are effective in decreasing alcohol use (Cornelius et al. 1995, 1997a, b) as well as depressive symptoms (Cornelius et al. 1997*a*,*b*; Roy 1998) in patients with comorbid alcoholism and depression. However, SSRIs may have a different effect depending on the subtype of alcoholic (Kranzler et al. 1996), and recent literature suggests that the SSRIs may be effective only in certain subtypes of depressed alcoholics (Pettinati et al. 2000*b*). A small open-label study (i.e., a study in which all participants receive the experimental treatment) has shown that SSRIs may be effective in patients with comorbid PTSD and alcoholism (Brady et al. 1995).

#### Antianxiety Medications

Several medications are available and effective in treating anxiety disorders. These include benzodiazepines; TCAs; SSRIs; and other serotonergic medications (i.e., medications that affect serotonin receptors), such as buspirone. Benzodiazepines are widely used for anxiety disorders, but some of their properties make their use controversial in patients with comorbid alcohol use disorders and anxiety disorders. For example, benzodiazepines have an abuse liability themselves and they can potentiate the motor and cognitive impairment associated with alcohol use. Despite these factors, little empirical evidence exists to suggest that these medications are unsafe for dual diagnosis patients. Clinically prudent treatment should include careful consideration of effective alternatives before prescribing benzodiazepines. If clinically indicated, benzodiazepines should be prescribed only after careful diagnosis, with close followup, including monitoring of abstinence and determination of continued need.

Both TCAs and SSRIs have been found effective in treating anxiety disorders, but their use in dually diagnosed patients has not been formally investigated. Because of their relatively minor side effects, the SSRIs often are the first line of treatment for anxiety disorders. Given recent evidence of their efficacy in treating alcohol use and depressive symptoms in dually diagnosed patients (Cornelius et al. 1995; 1997a, b), further study of these medications in this population seems warranted. Similarly, a small open-label study has shown that the SSRIs may be effective in ameliorating anxiety symptoms as well as alcohol consumption in patients with comorbid PTSD and alcoholism (Brady et al. 1995).

Other medications used for PTSD patients include the monoamine oxidase inhibitors (MAOIs) and anticonvulsants. MAOIs should be used with caution. It is imperative that patients taking these medications avoid foods and beverages containing the chemical tyramine— for example, beer on tap, red wines, liqueurs, overly ripe foods, salami, and aged cheeses. Patients actively abusing alcohol may be unable to adhere to this dietary restriction because of impaired judgment. The anticonvulsants are more promising because there is some evidence that they may be effective in treating anxiety disorders (Myrick et al. 2001). Their potential effectiveness as an alternative to benzodiazepines in the treatment of alcohol withdrawal suggests a role for them in the initiation of abstinence with comorbid patients. Although this application has only been reported anecdotally, this line of research is promising.

The serotonergic drug buspirone has been formally evaluated in several double-blind, placebo-controlled trials for patients with comorbid generalized anxiety disorder and alcohol dependence. Although several of these clinical studies suggested that buspirone reduced anxiety symptoms and one suggested that it reduced alcohol use, other studies reported that it had no effect in reducing anxiety or alcohol use (Myrick et al. 2001). These mixed results have left clinicians appropriately skeptical about the utility of this medication in this population.

#### Antipsychotic Medications

The first line of treatment for patients with schizophrenia or psychotic disorders is antipsychotic, or neuroleptic, medications. Because acute psychosis undermines a person’s ability to participate in social, community, and treatment activities, stabilizing these symptoms is usually the first priority in treatment. There are several classes of antipsychotic drugs with differing pharmacology, side effects, and receptor-binding affinity. No controlled studies exist to suggest that one type of antipsychotic is superior in treating the dually diagnosed patient. However, some general principles are known, based on research on the mechanisms of action of the different classes of antipsychotics, the underlying psychology and neurobiology of the disorders, and clinical experience. For example, the newer antipsychotics have been reported to better treat negative symptoms and have fewer movement disorder side effects (e.g., muscle spasms). Because negative symptoms may play a role in the etiology or maintenance of substance use disorders (i.e., people may use alcohol or other drugs to self-medicate the negative symptoms), these medications may be effective in treating the psychotic symptoms as well as alcohol use in this population. Additionally, this class of medications affects receptors, such as the serotonin receptors, that may play a role in alcohol abuse and dependence. Thus, they may be effective in treating alcohol use disorders as well as psychosis. Supporting these guidelines, a recent pilot study has suggested that clozapine may be effective in reducing symptoms of alcohol use disorders and schizophrenia in dually diagnosed patients (Drake et al. 2000).

Since formal research in this area is limited, a careful individual clinical history and an understanding of the issues specific to that particular population can help guide the choice for the most appropriate antipsychotic medication for each dually diagnosed patient. For example, it is important to consider the medication’s potential side effects and the patient’s history of medication compliance. It is also important to consider how alcohol may interact with the medication or exacerbate its effects. Highly sedating medications or medications that reduce the seizure threshold in a person concomitantly using alcohol may be problematic. Some patients’ psychotic symptoms cannot be stabilized because of medication noncompliance, preventing them from engaging in treatment. For such patients, it may be better to use medications that are injected intramuscularly and released slowly over time (Ziedonis and D’Avanzo 1998).

### Psychosocial Treatments for Comorbid Disorders

Recent developments in effective psychotherapy for alcohol use disorders, along with growing recognition that pharmacotherapy alone may not adequately address all the treatment requirements of comorbid patients, has led to the development of specialized psychotherapy for this population. The use of effective psychosocial treatments is particularly important among dually diagnosed patients for four reasons. First, there are some cases where pharmacotherapy may not be recommended (e.g., in alcoholic patients with anxiety disorders for whom taking benzodiazepines may be risky). Second, psychosocial treatments may be effective in treating functional deficits in patients with chronic psychiatric disorders, such as schizophrenia. Third, pharmacologic treatment enhanced with psychosocial approaches is important for patients with poor medication compliance. As mentioned above, even powerful psychopharmacologic treatments, such as disulfiram, are ineffective if patients do not take the medication. And fourth, effective psychosocial treatments are important for patients for whom early abstinence may be associated with a worsening of psychiatric symptoms, such as patients with PTSD who may experience anxiety with the cessation of alcohol use. The following sections review some of the issues surrounding psychotherapeutic treatment for comorbid disorders and the use of 12-step programs by dually diagnosed patients.

#### Psychotherapy

Psychotherapy research has led to the development of several treatments for patients with substance use disorders. These include an adaptation of psychodynamic approaches; cognitive behavioral techniques, such as relapse prevention and motivational enhancement therapy; and behavioral treatments, such as contingency management (Weiss and Najavits 1998). Some overarching principles and guidelines for the psychosocial treatment of patients with dual disorders have emerged from clinical descriptions and other reviews (Osher and Kofoed 1989; Drake et al. 1996). There seems to be some consensus that treatment should be viewed as occurring in stages and that immediate and short-term goals should be established and may not be identical. For example, although abstinence may be a long-term goal, patients with severe mental illness may not perceive their substance abuse to be a problem. The immediate goal of treatment with these patients may be stabilization of the psychiatric illness, followed by a discussion of their ambivalence about their alcohol use. Similarly, patients who achieve early abstinence from alcohol may need to be closely monitored for the emergence of symptoms of a psychiatric disorder, such as PTSD, whose presence may have been masked by their previous alcohol use.

Psychosocial treatments with dually diagnosed patients often involve the modification of standard techniques common in conventional primary substance abuse treatment settings or psychiatric treatment settings. For example, confrontation, a common and often effective technique in substance abuse treatment settings, may theoretically exacerbate psychotic thinking or suicidal ideation in patients with serious mental illness. In psychiatric treatment settings, laboratory testing for drug and alcohol use is often not routine, as it is in substance abuse treatment settings, and may be viewed by clinicians and patients as communicating distrust. Identifying and addressing issues not always connected with psychotherapy, such as homelessness and legal difficulties, may be the most beneficial aspect of the treatment. And clinicians who have received training for and understand both psychiatric and substance use disorders will most likely be successful in treating this group of patients, because they tend to have flexibility in their treatment approach and recognize that the goals of treatment may change as one disorder influences the other.

Two main approaches to specialized psychotherapy for patients with dual disorders have emerged. The treatment of patients with serious mental illnesses (i.e., patients with schizophrenia or schizoaffective disorder) and comorbid substance use disorders takes a different approach from the treatment of patients with anxiety disorders or mood disorders and comorbid substance use disorders. Patients with serious mental illnesses and substance use disorders are often the most difficult to treat in conventional substance abuse or psychiatric settings. Examples of treatments for these patients include: (1) a skills-based approach to therapy, (2) dual recovery therapy, (3) integrated assertive community treatment, and (4) money management therapy for patients with psychotic disorders and comorbid substance abuse disorders (Ziedonis and D’Avanzo 1998). These specialized psychotherapies have different focuses but share a common attempt to integrate and modify psychiatric and substance abuse treatment approaches to meet the needs of this population.

The approach to treating comorbid mood or anxiety disorders and alcohol use disorders is somewhat different, given the similarities and overlap among both the disorders and their treatments. For example, cognitive behavioral therapy has been shown to be effective in treating anxiety disorders and alcohol dependence separately and can be readily integrated for patients with comorbid alcoholism and anxiety disorders. Several psychotherapy components, such as relaxation training, stress management, and skills training, are emphasized in the treatment of both types of disorders. Because psychotherapy is usually tailored to the individual, one specialized area of focus may be the link between symptoms of anxiety and alcohol consumption. For example, techniques to identify and manage anxiety may also prevent relapse to alcohol use among comorbid patients.

#### 12-Step Programs

Twelve-step programs have been the focus of controversy in the substance abuse literature, particularly for the treatment of dually diagnosed patients. Although these programs may be beneficial to many people, those with severe mental illness may feel alienated. One factor associated with successful outcomes from self-help meetings is regular attendance, which may be influenced by a person’s level of motivation and early experience with 12-step treatment.

Referral to 12-step meetings can be beneficial and should be considered for many comorbid patients. Some areas to evaluate when considering whether to refer a patient to a 12-step group include the person’s level of motivation, barriers to attendance (including inability to relate to other people), previous experience with 12-step meetings, and the patient’s expectations and those of significant others. Special meetings for people with dual disorders exist in some geographical areas. Another possibility is to incorporate some of the aspects of the 12-step model into a specialized dual diagnosis program or an individually tailored psychotherapy.

## Summary and Conclusions

The chance of having a psychiatric disorder is significantly increased among people with alcohol dependence but not among those with alcohol abuse. Among people with alcohol dependence and a comorbid psychiatric disorder, the 1-year prevalence of major depressive disorder was estimated to be the highest of the mood disorders, and generalized anxiety disorder was most frequent among the anxiety disorders. People with comorbid psychiatric disorders are far more likely to receive treatment in specialized mental health services than those without comorbid disorders, although many people with comorbid psychiatric illness are not receiving specialized substance abuse treatment. Because these patients may receive treatment in mental health or substance abuse treatment facilities, it is important to implement and expand integrated services that address both addictive and psychiatric disorders. In addition, most research on treating alcohol use disorders has systematically excluded people with comorbid psychiatric disorders. The result is a wide gap between research and clinical realities. Further well-controlled research is needed to identify treatments, both psychotherapeutic and pharmaceutical, that are safe and effective for this population.
